# Obligate Insect Endosymbionts Exhibit Increased Ortholog Length Variation and Loss of Large Accessory Proteins Concurrent with Genome Shrinkage

**DOI:** 10.1093/gbe/evu055

**Published:** 2014-03-26

**Authors:** Laura J. Kenyon, Zakee L. Sabree

**Affiliations:** ^1^Department of Evolution, Ecology and Organismal Biology, The Ohio State University

**Keywords:** genome reduction, Flavobacteriaceae, Enterobacteriaceae, protein domains

## Abstract

Extreme genome reduction has been observed in obligate intracellular insect mutualists and is an assumed consequence of fixed, long-term host isolation. Rapid accumulation of mutations and pseudogenization of genes no longer vital for an intracellular lifestyle, followed by deletion of many genes, are factors that lead to genome reduction. Size reductions in individual genes due to small-scale deletions have also been implicated in contributing to overall genome shrinkage. Conserved protein functional domains are expected to exhibit low tolerance for mutations and therefore remain relatively unchanged throughout protein length reduction while nondomain regions, presumably under less selective pressures, would shorten. This hypothesis was tested using orthologous protein sets from the Flavobacteriaceae (phylum: Bacteroidetes) and Enterobacteriaceae (subphylum: Gammaproteobacteria) families, each of which includes some of the smallest known genomes. Upon examination of protein, functional domain, and nondomain region lengths, we found that proteins were not uniformly shrinking with genome reduction, but instead increased length variability and variability was observed in both the functional domain and nondomain regions. Additionally, as complete gene loss also contributes to overall genome shrinkage, we found that the largest proteins in the proteomes of nonhost-restricted bacteroidetial and gammaproteobacterial species often were inferred to be involved in secondary metabolic processes, extracellular sensing, or of unknown function. These proteins were absent in the proteomes of obligate insect endosymbionts. Therefore, loss of genes encoding large proteins not required for host-restricted lifestyles in obligate endosymbiont proteomes likely contributes to extreme genome reduction to a greater degree than gene shrinkage.

## Introduction

Currently, the majority of smallest known bacterial genomes are borne by host-restricted mutualists that reside within specialized host tissues. These bacteria are vertically inherited and resistant to in vitro cultivation, which is likely the result of their long term (e.g., tens to hundreds of millions of years), intracellular host associations (reviewed in Moran et al. 2008). The extreme reduction of these genomes has been the topic of many studies and there are several known attributing factors. Low effective population sizes and the occurrence of frequent bottlenecks in which bacteria are acquired by the offspring of their insect hosts every generation are prominent factors (Moran and Baumann 2000; Wernergreen 2002; Moran et al. 2008). These lead to an increase in the influence of genetic drift and a relaxation of selection for genes no longer vital in a stable habitat (Moran and Baumann 2000; Wernergreen 2002). Thus, during the initial lifestyle change from free-living to endosymbiotic, genes that are not essential for maintaining the endosymbiotic relationship quickly acquire mutations, become pseudogenized, and are lost. The environmental and nutritional stability within host cells precludes the necessity for many of the enzymes that provide metabolic flexibility and resilience to diverse conditions experienced by related, free-living taxa. In addition, the opportunities to acquire new genetic material from other bacteria are limited due to isolation within host tissues. Genome reduction is impacted by relaxed selection and loss of DNA repair and recombination mechanisms that contribute to the high mutation rate observed in these tiny genomes (Moran 1996). Additionally, there is a mutational bias toward deletions observed in bacteria (Mira et al. 2001). Yet another factor contributing to the initial genome reduction after a lifestyle change could be the loss of accessory (nonessential) proteins in the early evolution of genome reduction due to selection for this loss, rather than to drift (Lee and Marx 2012). These factors together likely lead to the extreme reduction in genome size witnessed in insect endosymbionts.

Another potential factor influencing the genome size of bacterial insect endosymbionts is protein length. Previous research has suggested that there may be selection for the smallest possible proteins that are still able to maintain function in bacterial cells, as smaller proteins would be less metabolically expensive (i.e., Wang et al. 2011). This pressure would theoretically be highest in smaller genomes, such as those in obligate endosymbionts. In fact, Charles et al. (1999) showed that many orthologous proteins were shorter in the obligate endosymbiont of aphids, *Buchnera aphidicola*, than in *Escherichia coli*, a free-living relative. Similarly, Wang et al. (2011) showed that proteins are decreasing in length in bacterial lineages. However, others have noted that this may not be the case. For instance, Kuo et al. (2009) pointed out that there is no strong association between genome size and doubling time of bacteria and that the obligate endosymbionts (with the smallest genomes) do not have a lifestyle that promotes selection for rapidly dividing cells. In fact, recent evidence suggests that cell division in endosymbionts, for example, *Rhizobium* in legumes (Mergaert et al. 2006) and SOPE in cereal weevils (reviewed in Login and Heddi 2012), might be under host control. Additionally, many insect endosymbionts have reduced ability to undergo cytokinesis and are often present as polyploids, with many chromosome copies contained within a single gigantic cell (reviewed in Komaki and Ishikawa 1999; Komaki and Ishikawa 2000; Baumann 2005). Thus, selection for rapidly dividing cells in endosymbionts appears to be absent or at best extremely weak.

Previous work has suggested differential selection within protein sequences depending on the necessity of specific residues for protein function. Specifically, protein regions that perform catalytic functions (i.e., “domains”) are under greater selection than portions of the proteins that do not (i.e., “linker regions”) (i.e., Wang et al. 2007; Wang and Caetano-Anollés 2009). Wang et al. (2011) found that across Bacteria, as proteome diversity (i.e., number of protein-coding genes) decreased, the size of the proteins decreased, the domain lengths stayed the same, and the linker lengths decreased. If proteins were getting shorter, we would expect domains to vary little in length, due to selective pressures, and the linkers to be degraded. Using genome size as an indicator of proteome diversity, orthologous protein sets were identified in two distinct bacterial lineages that included taxa exhibiting extreme genome reduction. Orthologs with shared functional domain architectures were determined and the hypothesis that, in highly reduced genomes, linker lengths shortened in length while domain lengths remained fixed in orthologs was examined.

## Materials and Methods

### Selection of Taxa Used in the Study

Orthologs from two bacterial families Flavobacteriaceae (phylum: Bacteroidetes) and Enterobacteriaceae (subphylum: Gammaproteobacteria) that contained both obligate insect endosymbionts (OIE) and nonobligate insect endosymbionts (nonOIE) with complete genome sequences available were identified and used in this study. These two families were chosen as the focal groups because they contain the majority of the known OIEs with highly reduced genomes that are fully sequenced and annotated, and the genera included in our analyses were limited to those for which at least two complete genomes for species of each genera (e.g., *Blattabacterium sp.* BPLAN and *Blattabacterium sp.* Bge) were available. While OIEs are found in other families, that is, *Wolbachia* in Alphaproteobacteria and *Tremblaya* in Betaproteobacteria, potential biases associated with having few OIE genera per phylum or subphylum to compare with nonOIE relatives were avoided. Finally, similar numbers of species representing OIE and nonOIE lifestyles within each of the two families were chosen in order to make statistically significant comparisons; 20 OIE and 27 nonOIE Enterobacteriaceae genomes and 11 OIE and 11 nonOIE Flavobacteriaceae genomes were included (supplementary material S1, Supplementary Material online). Habitats of selected nonOIE species included nonobligately host-associated and free-living taxa, and they were chosen based on their diversity of habitats (see supplementary material S10, Supplementary Material online). Proteomes were downloaded from the National Center for Biotechnology Information “nr” database (http://www.ncbi.nlm.nih.gov, last accessed March 28, 2014) using custom, in-house informatics tools.

### Detection of Orthologous Proteins and Domain Regions

OrthoMCL (Chen et al. 2006) and custom Perl scripts were used to identify orthologous proteins, and the Pfam web interface (Punta et al. 2012) was used (*e*-value = 1.0, default) to search the Pfam database for domain identification. Orthologous proteins that shared identical domain architecture (i.e., number and annotation of domains), protein annotation (if available), and had standard deviations for total protein lengths of less than 30 were retained. Preliminary inspection of the protein lengths revealed that orthologous groups with standard deviations of more than 30 were almost consistent due to gene fusions in one of the bacterial lineages. As including gene fusion events in this study could be misleading, these were removed. Proteins with multiple domains that were overlapping were not included unless one domain entirely encompassed a smaller domain, in which case the length of the larger domain was used. Upon visual inspection of individual MAFFT alignments of orthologous protein sets (Katoh and Standley 2013), all outliers due to annotation errors were edited or not included (listed in supplementary material S7, Supplementary Material online). Pfam was used to identify functional domain region lengths, and linker regions were protein regions not annotated as part of a functional domain. Linker length in each protein was determined by subtracting the sum of the functional domain lengths from the total protein length for each protein (supplementary material S8, Supplementary Material online). Statistical analyses were performed within R (http://www.r-project.org, last accessed March 28, 2014). RAxML was used to generate maximum likelihood trees for each family using concatenated orthologous protein sequences within the CIPRES portal using the cpREV protein matrix (Adachi et al. 2000; Stamatakis et al. 2008; Miller et al. 2010).

### Calculating Gap Frequencies in Orthologous Protein Alignments

In order to evaluate which region(s) (terminuses vs. central portions) of the proteins tended to undergo more insertion or deletion mutations (i.e., mutations that would affect protein length), a custom Python (http://python.org, last accessed March 28, 2014) script that divided each protein in an alignment into 10 equal sections plus an 11th section when the alignment could not be divided evenly (which contained the remainder of residues unable to fit into the first 10 sections) and counted the gaps (“-”) present in each section was implemented. Alignments were performed in ClustalW (Larkin et al. 2007), MAFFT (parameters: E-INS-I and L-INS-I algorithms), and MUSCLE (Edgar 2004) for each orthologous protein set. Each alignment algorithm was run in triplicate with randomly shuffled sequences for each run, to avoid bias based on sequence input order, and averages of these three runs are reported.

### Evaluation of the Maximum Protein Length in Gammaproteobacteria and Bacteroidetes Proteomes

Proteomes from 79 Gammaproteobacteria and 42 Bacteroidetes species occupying different habitats (including species outside of the two focal families used in the previous ortholog domain and linker analyses) were used for evaluation of maximum protein lengths and total proteomes were examined (supplementary material S6, Supplementary Material online). Representative proteomes were downloaded from the NCBI database and “infoseq” (EMBOSS, Rice et al. 2000) was used to determine protein lengths. A custom Python script extracted protein sequences with lengths longer than the desired cut-offs. BLASTP searches were performed with selected proteins of the Clusters of Orthologous Groups (COG) database (Tatusov et al. 1997) to assign these proteins to functional groups. The KAAS - KEGG Automatic Annotation Server (Moriya et al. 2007) was also used to assign the proteins to KEGG (Kyoto Encyclopedia of Genes and Genomes) Orthology groups (http://www.kegg.jp, last accessed March 28, 2014).

## Results

### Orthologous Proteins and Bacterial Lineages Used in This Study

Eighty-two and 71 orthologs were drawn from the proteomes of 22 Flavobacteriaceae and 47 Enterobacteriaceae species, respectively, to determine whether analogous or phylum-specific trends related to bacterial lifestyle could be detected (supplementary material S1, Supplementary Material online). Genome sizes for these taxa spanned 0.24–6.1 Mb and were classified based on their habitat (e.g., OIE or nonOIE). Orthologous protein sets were used to test hypotheses about the impact of bacterial species lifestyle on genome reduction and protein length in two bacterial lineages.

### Obligate Insect Endosymbiont Orthologs Exhibit Increased Protein Length Variation

**Fig. 1.—:**
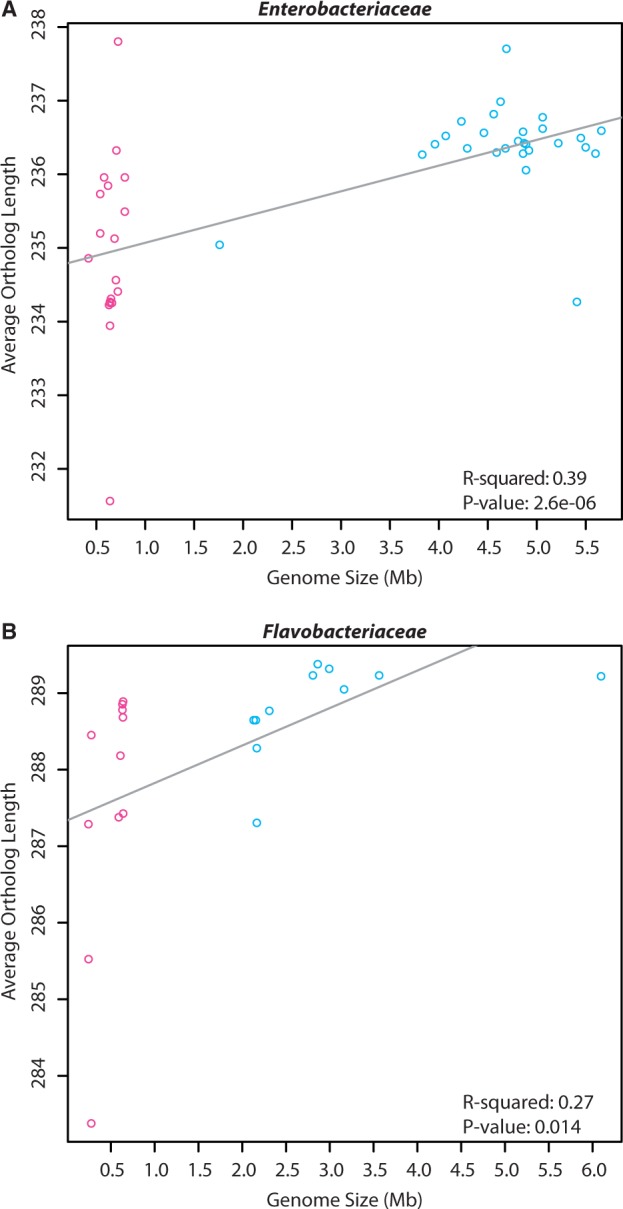
Enterobacteriaceae (*a*) and Flavobacteriaceae (*b*) average orthologous protein lengths as a function of genome size. The variance for the OIE data points is 1.56 and 2.91 for Enterobacteriaceae and Flavobacteriaceae, respectively. For the nonOIE it is 0.35 and 0.38, respectively. *Y*-axis values are the average ortholog lengths in number of amino acid residues.

Evidence from previous studies has suggested that protein length decreases with total CDS length (i.e., Wang et al. 2011) and orthologous proteins of OIE genomes are smaller than that of nonOIE relatives (i.e., Charles et al. 1999). The hypothesis that average protein length decreases as genome size decreases in two divergent bacterial lineages was tested. As total CDS length was highly correlated with genome size in both families (*P* < 2.2e−16, *R*^2 ^> 0.97), only genome size was used. On average, OIE orthologs decreased in length and exhibited greater total length variation in both Enterobacteriaceae (*P* = 2.6e−6, *R*^2 ^= 0.39) (fig. 1*a*) and Flavobacteriaceae (*P* = 0.014, *R*^2 ^= 0.27) (fig. 1*b*) lineages. The increased variability of average OIE protein lengths likely reflects elevated mutation rates commonly observed in endosymbiotic lineages (i.e., Moran et al. 2008). Additionally, OIE lineages had longer branch lengths than nonOIE lineages in maximum-likelihood trees based on concatenated orthologs (supplementary material S2*a* and *b*, Supplementary Material online) supporting greater sequence divergence in obligate host-associated taxa.

**Fig. 2.—:**
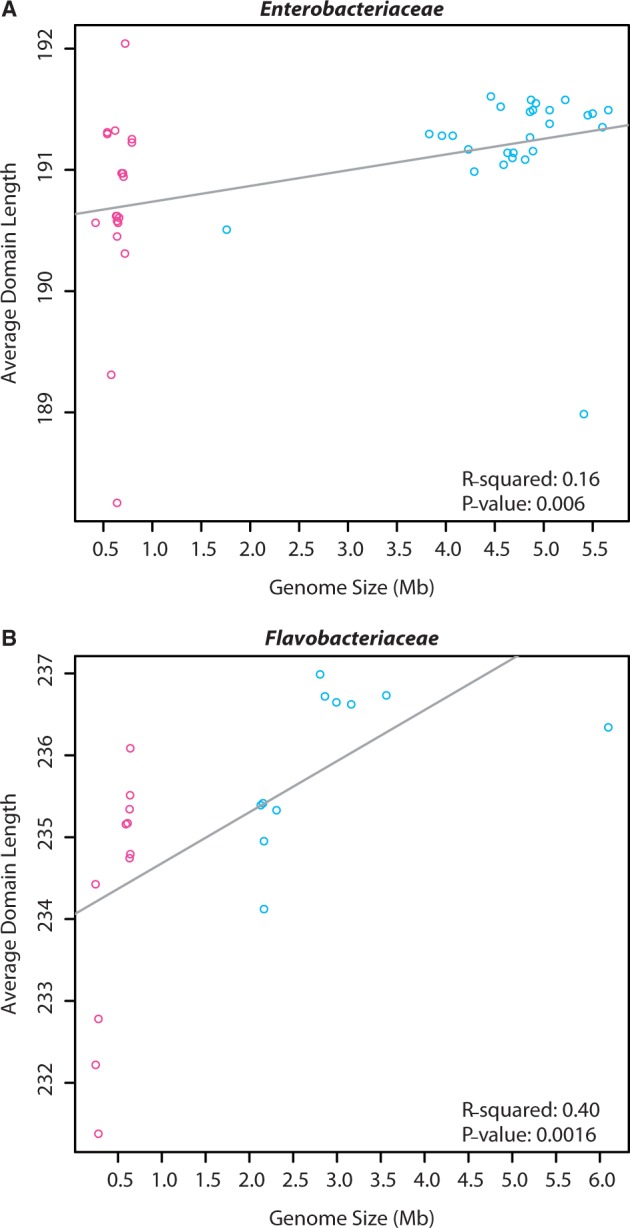
Enterobacteriaceae (*a*) and Flavobacteriaceae (*b*) average orthologous domain lengths as a function of genome size. The variance for the OIE data points is 0.63 and 2.29 for Enterobacteriaceae and Flavobacteriaceae, respectively. For the nonOIE it is 0.26 and 0.87, respectively. *Y*-axis values are the average ortholog lengths in number of amino acid residues.

**Fig. 3.—:**
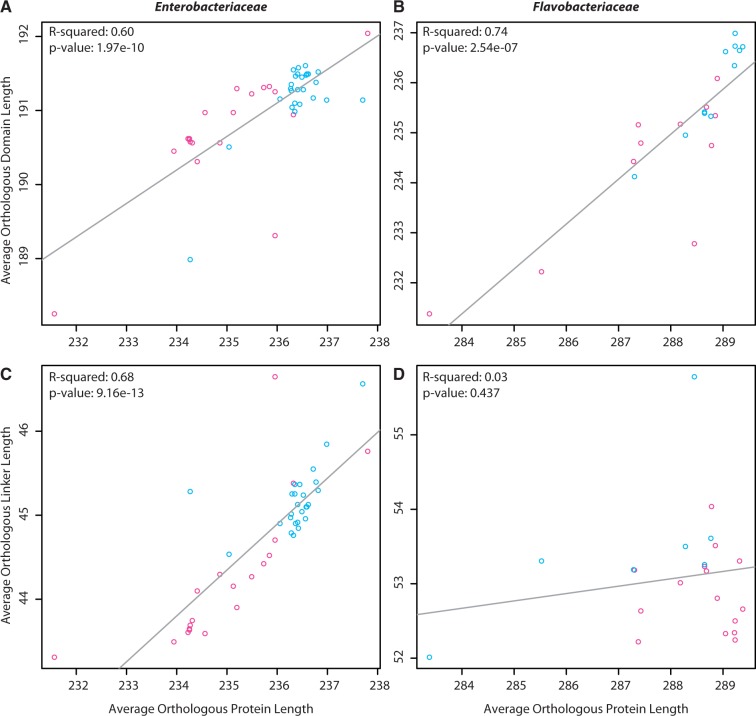
Average orthologous domain lengths (*a*, *b*) and average orthologous linker lengths (*c*, *d*) as functions of average orthologous protein lengths for Enterobacteriaceae and Flavobacteriaceae, respectively. *X*- and *y*-axis values are the lengths in number of amino acid residues.

If functional domains are under greater selection than linker regions due to their role in enzyme activity, then, as genome sizes decrease, average protein lengths were expected to decrease while average functional domain lengths remained fixed. However, average orthologous domain lengths were positively correlated (*P* < 0.05) with genome size in both families (fig. 2*a* and *b*), and the variance of average OIE domain lengths was greater than nonOIE average domain lengths. Both bacterial lineages exhibited significant positive correlations between the average total protein lengths and the average domain lengths (fig. 3*a* and *c*). Additionally, several domains showed significantly (*t*-test *P* < 0.05) greater lengths in the OIE proteins than in the nonOIE proteins. Therefore, domain lengths did not remain fixed as genome length varied in either lineage (fig. 2*a* and *b*). Instead, these data show that domain lengths in OIE proteins are more variable than in nonOIE orthologs.

**Fig. 4.—:**
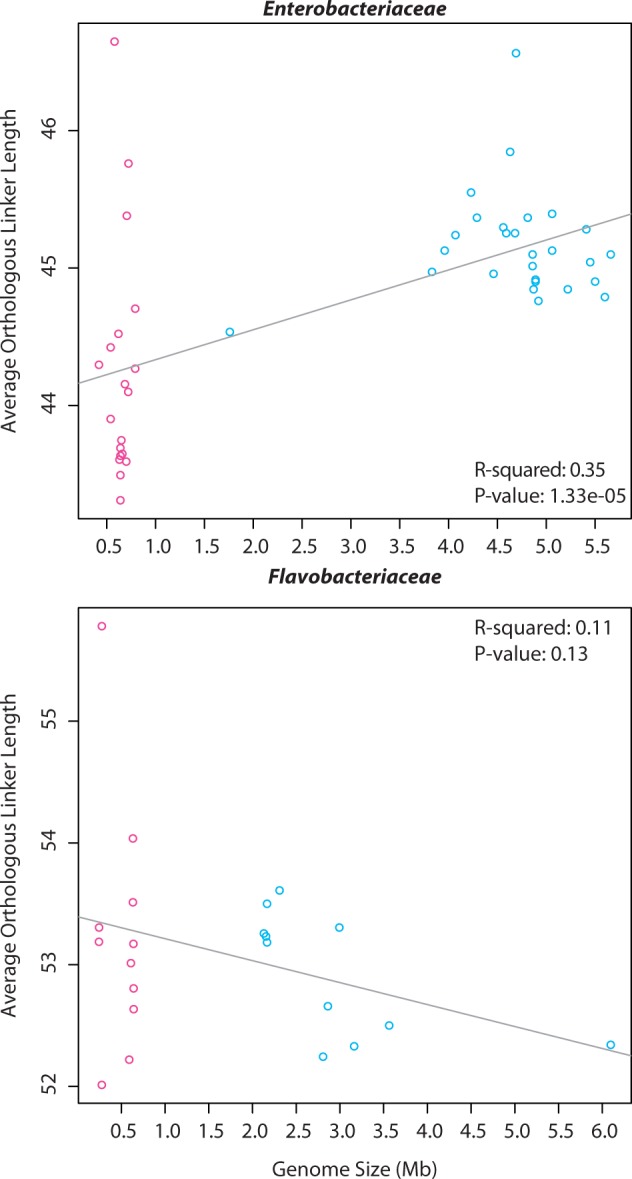
Enterobacteriaceae (*a*) and Flavobacteriaceae (*b*) average orthologous linker lengths as a function of genome size. *y*-axis values are the average ortholog lengths in number of amino acid residues.

The expectation that protein linker regions are less sensitive to indel mutations, due to lower functional constraints, and exhibit greater length variation than functional domains in reduced genomes was explored. Positive correlations between average linker length and both genome size (fig. 4*a*) and average protein length (fig. 3*b*) were observed for Enterobacteriaceae orthologs, with OIE protein linker lengths differing by 4 amino acids and average total protein lengths differing by ∼7 amino acids. These results suggest that, on average, both linker and domain length variations are contributing to total protein length variation. Average linker lengths for Flavobacteriaceae orthologs did not vary significantly with genome size (fig. 4*b*) or total protein length (fig. 3*d*).

Length distributions for orthologous protein sets were investigated individually to detect whether OIE proteins were more variable in length when compared with the nonOIE proteins and to exclude the possibility of a few outliers biasing the trends observed when using averages. Although the genomes of OIE species were generally smaller (<1 Mb) than nonOIE species (>1 Mb), OIE orthologs were not uniformly smaller, but instead more variable in length than their orthologous counterparts in nonOIE species. In fact, over half of the OIE orthologous protein sets in both families were, on average, longer than orthologs in nonOIE species and the distributions of ortholog lengths for >75% of the OIE orthologous protein sets had ranges that were larger than (excluding outliers) and significantly different (*t*-test and Mann–Whitney *P* < 0.001) from nonOIE orthologs (tables 1 and 2). Additional metrics of differences (e.g., standard deviations and interquartile ranges) also showed significantly greater (*P* < 0.001, table 2) length variation for OIE orthologs versus nonOIE orthologs for nearly all orthologous protein sets for both bacterial families.

**Table 1:** OIE (obligate insect endosymbiont) Protein Lengths Vary More Frequently than nonOIE (lifestyle other than OIE) Orthologs

Length Variability	Range	SD	IQR
Flavobacteriaceae			
No variability	5 (6.10%)	2 (2.44%)	5 (6.10%)
Variable in both	4 (4.88%)	0 (0.00%)	4 (4.88%)
nonOIE more variable	11 (13.41%)	14 (17.07%)	16 (19.51%)
OIE more variable	62 (75.61%)	66 (80.49%)	57 (69.51%)
Enterobacteriaceae			
No variability	11 (15.49%)	1 (1.41%)	11 (15.49%)
Variable in both	1 (1.41%)	0 (0.00%)	1 (1.41%)
nonOIE more variable	4 (5.63%)	10 (14.08%)	5 (7.04%)
OIE more variable	55 (77.46%)	60 (84.51%)	54 (76.06%)

Note.—SD, standard deviation; IQR, interquartile range. "Range" indicates the range with the exclusion of outliers (as determined by box and whisker plots in R). The values reported represent the number of orthologous protein sets and the proportions of the total orthologous protein sets used are parenthesized.

**Table 2:** OIE (obligate insect endosymbiont) Proteins Exhibit Significantly Greater Length Variability than nonOIE (lifestyle other than OIE) Orthologs

	Range	SD	IQR
Flavobacteriaceae			
nonOIE average	3.87	2.48	2.80
OIE average	12.15	6.68	5.82
*t*-test *P* value (nonOIE vs. OIE)	2.20E−06	1.43E−06	0.00033
Mann–Whitney *P* value (nonOIE vs. OIE)	8.74E−09	2.00E−09	2.98E−06
Enterobacteriaceae			
nonOIE average	1.87	1.68	0.70
OIE average	7.04	3.07	3.06
*t*-test *P* value (nonOIE vs. OIE)	1.59E−06	0.00016	6.99E−07
Mann–Whitney *P* value (nonOIE vs. OIE)	1.12E−10	4.50E−09	4.71E−11

Note.—SD, standard deviation; IQR interquartile range. Range represents averages protein lengths for ortholog sets to the exclusion of outliers (as determined by box and whisker plots in R) within each orthologous protein set.

**Fig. 5.—:**
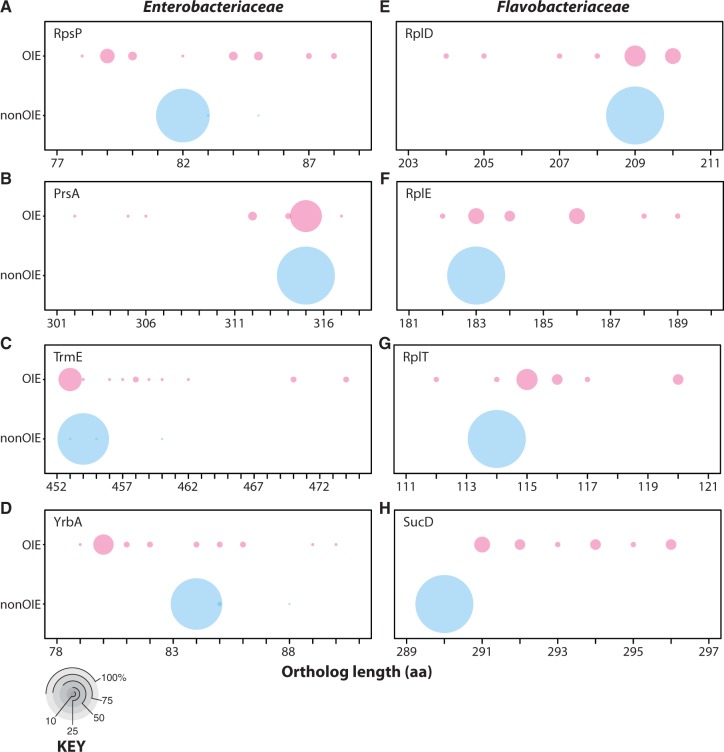
Bubble plots of orthologous proteins representing proteins with OIE lengths more variable than nonOIE lengths. The *x*-axis depicts protein length in amino acid residues. The bubbles are scaled by the number of proteomes with a protein at a particular length out of the total number of proteomes. Each table is labeled with the orthologous protein set it represents. The key shows what various proportions look like as bubbles. *X*-axis values are the ortholog lengths in number of amino acid residues.

Bubble plots for each orthologous set were generated to illustrate these differences (fig. 5*a*–*h* and supplementary material S3, Supplementary Material online). Finally, the variability of domain versus linker or total protein lengths were compared for each individual orthologous protein set, with the prediction that linker lengths would have greater variability than domain lengths due to greater selection upon functional domains, but no significant differences between the standard deviations of the overall protein, domain, and linker lengths for the OIE and nonOIE orthologs for either family was observed (supplementary material S4, Supplementary Material online), which does not support the prediction.

### Indel Mutation Bias at Protein Terminuses

Previous work has suggested that protein mutations are more likely to occur at the N- or C-terminuses rather than in the central core of the protein (Charles et al. 1999; Kurland et al. 2007); thus, the hypothesis that mutations would occur at the terminuses, irrespective of domain location was tested. In both the Enterobacteriaceae and Flavobacteriaceae orthologs studied, most of the gaps occur at the terminuses when using four different alignment algorithms and three different sequence orders for each algorithm (supplementary material S9, Supplementary Material online).

### Large Proteins Involved in Secondary Cellular Processes Are Absent in OIE Proteomes

**Fig. 6.—:**
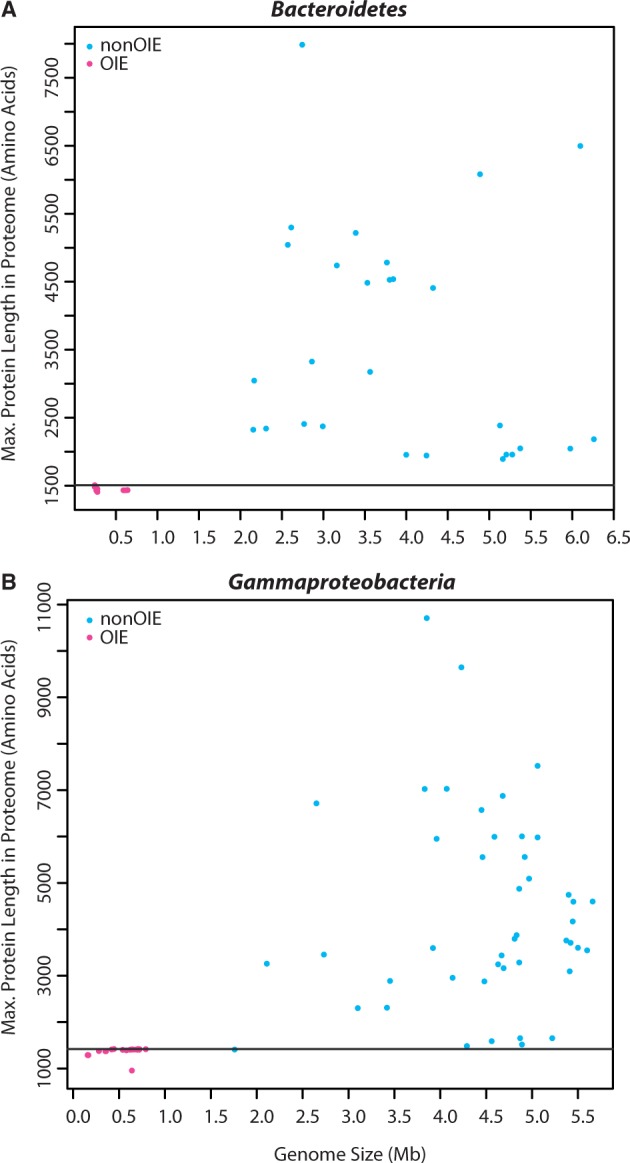
Plots showing the maximum protein lengths in the proteomes of 42 Bacteroidetes species (*a*) and 79 Gammaproteobacteria species (*b*). In each plot, a reference line is drawn at the cut-off for the maximum protein length found in OIE species (y = 1,507 aa in Bacteroidetes, y = 1,420 aa in Gammaproteobacteria). *Y*-axis values are protein lengths in number of amino acid residues.

**Fig. 7.—:**
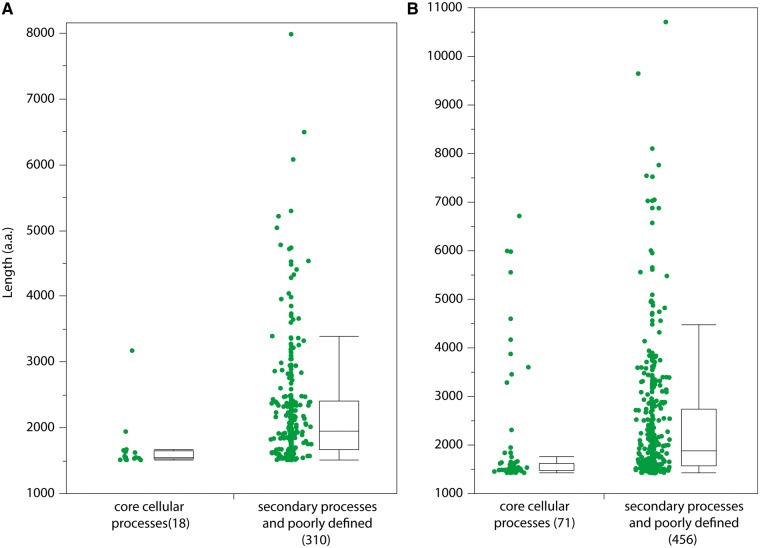
Obligate insect endosymbionts have lost large proteins nonessential for host-restricted mutualisms. Proteins from free-living and nonobligate host-associated Bacteroidetes (*a*) and Enterobacteriaceae (*b*) species that are longer than the longest obligate insect endosymbiont proteins are grouped into those involved in key cellular functions (i.e., core cellular processes) or secondary metabolic processes/pathogenesis/unknown functions (i.e., secondary processes and poorly defined). Parenthesized values represent number of proteins that were assigned to each category. Length on *y* axis is number of amino acid (aa) residues.

Examination of maximum protein lengths within Gammaproteobacteria and Bacteroidetes OIE proteomes revealed that none were larger than 1,507 amino acids long while the nonOIE members of these groups had maximum protein lengths ranging from 1,408 aa (*Serratia symbiotica* str. “*Cinara* cedri,” RNA Polymerase β′ subunit, NCBI accession# AEW44827) up to 10,708 aa (*Xanthomonas albilineans* non-ribosomal peptide synthase; NCBI accession# YP_003375559; fig. 6). OIE genomes are enriched in genes encoding enzymes involved in central metabolic processes, namely ATP and nutrient-generating pathways, while lacking those genes encoding for enzymes involved in secondary metabolism and cell division. To this end, the annotated functions for all enzymes longer than the longest OIE proteins (heretofore referred to as “large proteins”) for the two bacterial groups (e.g., >1,420 aa for Gammaproteobacteria and >1,507 aa for Bacteroidetes) were investigated to determine what proportion of these proteins could be classified as participating in 1) core cellular processes (e.g., glycolysis and energy production; amino acid and vitamin biosynthesis; DNA replication, transcription, and translation), or 2) secondary/“conditional” processes (e.g., toxin and self-defense compound production; extracellular sensing) or unknown functions. Large proteins involved in core cellular processes were generally the same length and <1,600 residues long, while those assigned to secondary processes or of unknown function exceeded 1,600 residues in length and they were, on average, significantly longer (Mann–Whitney test: gammaproteobacterial: χ^2 ^= 73.9, dF = 1, *P* = <0.0001; bacteroidial: χ^2 ^= 22.6, dF = 1, *P* = <0.0001) than proteins assigned to the core cellular processes (fig. 7*a* and *b*). The relatively few large proteins assigned to the core processes category were involved in DNA replication (e.g., bifunctional DNA polymerase III subunit alpha/DNA polymerase III, epsilon subunit), RNA transcription (e.g., RNA polymerase β′ subunit), recombination (e.g., exodeoxyribonuclease V and helicase), and purine assembly (e.g., phosphoribosylformyl-glycineamide synthetase). Shorter orthologs of these proteins (as defined by “BLASTP” alignment resulting in >65% amino acid sequence identity over >80% of the length of OIE proteins) were detected in OIE proteomes. 87–96% of the large proteins are involved in secondary processes, of which 18% and 48% of the gammaproteobacterial and bacteroidial large proteins, respectively, were conserved hypothetical proteins up to 7,986 residues long. Intracellular invasion and survival, polyketide and nonribosomal peptide synthesis, cellulose degradation, radical oxygen species scavenging, and biofilm formation were among the many cellular operations large proteins categorized among the secondary processes engaged in.

## Discussion

### Increased Length Variability, and Not Uniform Shrinkage, Typifies Endosymbiont Orthologs

Based on previous observations that protein length reduction is positively correlated with proteome size (i.e., Kurland et al. 2007; Wang et al. 2011) and protein lengths for obligate insect endosymbiotic lineages (OIE) are shorter than seen in their free-living relatives (i.e., Charles et al. 1999), the hypothesis that protein length decreases with genome size was tested in two divergent lineages of bacteria. OIE orthologs were more variable in length, and not uniformly smaller, than those in their free-living relatives (nonOIE) for both bacterial families. Genome size and average ortholog lengths were significantly correlated for the Flavobacteriaceae and Enterobacteriaceae, but the correlation coefficients were low (*R*^2 ^= <0.40) and several average OIE ortholog lengths were larger than averages of nonOIE ortholog lengths. In both families, the variance of average OIE ortholog lengths was greater than nonOIE orthologs.

Patterns of individual orthologous protein length variance reflected overall average ortholog length variance in that OIE ortholog lengths varied more than nonOIE ortholog lengths. Examining ortholog length distributions for individual orthologous protein sets excluded the possibility that length distribution patterns distinct from those determined using protein lengths averaged across all proteins per habitat, per lineage were masked. For example, some orthologous protein sets may have proteins that are getting smaller with genome reduction, but overall average lengths would not reflect this due to the possible presence of proteins in other orthologous protein sets getting larger. However, most of the orthologs did not uniformly decrease in size with genome reduction since the average lengths of the OIE proteins were larger than the averages of the nonOIE proteins for over half of the orthologous protein sets in both bacterial families.

Increased ortholog length variability, rather than ortholog length reduction in OIE taxa, did not reflect initial predictions but are not without precedent. For instance, although Kurland et al. (2007) suggested that there is pressure for minimal size of proteins in Archaea and Bacteria, they also found that there was only a weak positive correlation between average protein length and genome size. Further, they noted that the inclusion of pseudogenes could make it appear as though proteins are getting smaller, when, in actuality, many of them are no longer functional (Kurland et al. 2007). This is perhaps the case in previous studies that have detected a decrease in ortholog length with genome size, especially considering the increased pseudogenization of genes that occurs during the evolution of obligate mutualisms between insects and endosymbionts. The data examined here only included orthologous proteins with the same domain architecture, to the exclusion of annotated pseudogenes. Also, previous work has suggested that there may be selection pressures in bacteria for minimally sized proteins in large, complex proteomes with large population sizes; however, this pressure is likely weak in OIE lineages due to their simple, small proteomes and small effective population sizes (Kurland et al. 2007). Thus, the extreme genome reduction observed in OIEs is not attributed to selection for smaller proteins, but due to entire gene loss due to the loss of DNA repair mechanisms, lack of opportunities to acquire new genetic information, relaxed selection on proteins no longer necessary in their stable, intracellular environments, and the bottlenecks experienced every generation (i.e., Wernergreen and Moran 1999; Wernergreen 2002). In fact, Charles et al. (1999) noted that although they found that *Buchnera* genes were significantly smaller than *E. coli* genes (85 protein genes examined), this reduction in protein lengths could only account for <0.005% of the genome size reduction in *Buchnera*, and thus it is unlikely that ortholog length reduction decreases the energetic cost of protein synthesis enough to be strongly selected for.

Further experimental work is needed to address how the observed length variability in OIE orthologs impacts their biosynthetic functions within their specific host contexts and whether adaptive advantages exist. Some evidence based on well-studied proteins such as the lac repressor, T4 lysozyme, λ Cro, λ repressor, and *Staphylococcus* nuclease suggests that many amino acid substitutions have neutral or nearly neutral impacts on the structural stability of proteins (Kurland 1992). But it is unclear what impact these indels have on the structural stability of OIE proteins. Lambert and Moran (1998) showed that the secondary structures of Domain I of the 16S rRNA in OIE genomes was less stable than the nonOIE counterparts and the OIE 16S rRNA stabilities varied more than the nonOIE stabilities (which showed very little variation). Thus, if proteins exhibit similar patterns as the 16S rRNA, it seems likely that the increased variability observed in OIE orthologous protein lengths may reflect decreased stability in these proteins. Supporting this hypothesis is the observed overexpression of the chaperone GroEL in *Buchnera* cells, perhaps reflecting the increased need for protein folding error correction by the chaperone due to there being more misfolded proteins (i.e., Baumann et al. 1996; Moran 1996; Fares et al. 2004).

### No Sacred Ground: Functional Domains and Linker Regions of OIE Proteins Both Exhibit Elevated Length Variation

The variability observed in OIE ortholog lengths is due to variation in both the linker and the domain regions of the orthologs, contrary to the expectation that domain lengths would remain fixed throughout genome reduction. Protein linker regions are assumed to lack a stable tertiary structure and are involved in binding and recognition of a diversity of molecules to assist in complex formation, whereas the domain regions are involved in reaction catalysis and thus functionally conserved evolutionary units (Kurland et al. 2007; Wang et al. 2011). Conserved regions were predicted to remain unchanged during the extreme genome reduction as seen in OIE species. However, the average lengths of domains varied with genome size and there were significant positive correlations between the average domain and average ortholog lengths, suggesting that domain lengths were not fixed. As observed with overall ortholog lengths, domain lengths were also more variable in OIE orthologs than in nonOIE orthologs and were not uniformly smaller in OIE orthologs. Ortholog linker lengths varied, as expected, in Enterobacteriaceae with positive correlations between average linker lengths and genome size, and average linkers lengths and average ortholog lengths. Although the two bacterial families chosen were expected to show converging patterns of protein length evolution, similar patterns were not observed in orthologs of Flavobacteriaceae taxa, suggesting that the variation observed in overall ortholog lengths was due to variation in the domain regions, not the linker regions. This could be due to several factors. For instance, the divergence times between the OIE and nonOIE organisms in each of the families are not the same and thus we could be looking at different snapshots of evolutionary time when comparing the two families (and therefore witnessing slightly different patterns). Alternatively, as more orthologs were used in the Flavobacteriaceae studies at the cost of studying fewer organisms, this could somehow be influencing the results. Finally, perhaps the same evolutionary rules do not apply to both families: within Enterobacteriaceae we see the patterns we might expect with protein length decreasing as genome size decreases, but this is not the case in Flavobacteriaceae due to unknown differences.

Because the orthologous domain lengths vary more than expected (in that no variation was expected), the hypothesis that more indel mutations occur at the terminuses of the proteins, rather than the central core, irrespective of domain location was tested. This has been suggested in previous studies (i.e., Charles et al. 1999; Kurland et al. 2007) and was observed in this study. Previous work has suggested that amino acid substitutions with the largest affect on the physical stability of the protein are usually not in the active sites but rather the hydrophobic core of the protein (reviewed in Kurland 1992). In addition to affecting protein stability significantly, mutations in the central core might simply be less likely than mutations of the peripherally exposed residues (Chothia and Gough 2009). Furthermore, Chothia and Gough (2009) suggested that mutations in areas outside of the core are four times more likely than those inside the core. Thus, the lack of conservation of domain lengths observed might be because the domains are not always located in the central core of the proteins, and thus not always the regions that affect protein stability significantly. Another potential cause for the observed domain length variance in OIE proteins could be that some of the OIE genomes are still in the process of reduction and not quite at their minimal genome yet. In order words, this data may reflect varying levels of genome reduction within the OIEs, thus leading to varying domain lengths. Although less likely due to the strong bottlenecks experienced by OIEs at each generation, domains may be under positive selection where they exhibit dramatically longer or shorter lengths, but further evidence from molecular or enzymological investigations would lend support to this hypothesis.

Protein, domain, and linker lengths appear to vary among OIE proteomes; but what is unclear is whether an amino acid length difference of only one or two residues would impact the function, and therefore evolution, of the protein. While some single mutations cause no measurable changes in fitness, others can change the function completely or make the protein nonfunctional. Only 30% of amino acid mutations in *TEM1* β-lactamase lead to a decrease in fitness (reviewed in Soskine and Tawfik 2010), yet mutation analyses in other reported loci reveal a range of effects of amino acid substitutions and indels on protein function. Single amino acid changes in the *Salmonella* mannose-specific type I fimbrial adhesin protein FimH has been shown to alter its mannose binding ability and dramatically impact serovar pathogenicity (Kisiela et al. 2012). Similarly, FimH in *E. coli* uropathogenic strains have a structural point mutation in *fimH* which encodes for a protein with elevated monomannose receptor binding affinity in the bladder (Sokurenko et al. 2004). Another example of how the addition of a few amino acids to a protein can lead to a new function is evidenced in the GroEL generated by *Enterococcus aerogenes* symbionts of predatory larval antlions (family Myrmelentidae, order Neuroptera). While this GroEL functions as a protein-folding chaperone, it also is employed by the host as a toxin that is secreted in its saliva that paralyzes the prey. Toxicity of the GroEL produced by the antlion symbiont has been linked to four residues, which are not found in nontoxic GroEL produced by related enterobacteria (Yoshida et al. 2001). These few examples provide some evidence that relatively few residue changes can significantly alter protein function.

### Use It or Lose It: The Loss of Genes Encoding Large, Nonessential Proteins Contributes to Genome Shrinkage in Endosymbiotic Lineages

Increased gene length variability, and not uniform length reduction relative to genome size, was observed for OIE orthologs, which suggests that their contribution to overall genome size is minimal.

Gene deletions likely have a greater impact on overall genome size reduction than genic indels. Genes encoding functions not involved in core cellular processes like cell maintenance and central metabolism are often absent in the genomes of obligate intracellular taxa and the loss of these genes, especially if they are generally longer in length, would contribute to rapid overall genome size reduction. Therefore, nonOIE gammaproteobacterial and bacteroidial proteins longer than the longest OIE proteins were functionally categorized (e.g., core cellular processes or secondary processes/unknown function) to determine 1) how these proteins were distributed between these categories and 2) whether these proteins had smaller orthologs among the OIE proteins. For both bacterial groups, the majority of large proteins were >1,600 residues long and categorized as poorly defined or involved in secondary processes (fig. 7*a* and *b*), and these proteins were, on average, significantly longer than those involved in core cellular processes. Overall, few of the large nonOIE proteins had orthologs in the OIE proteome, and these were limited to enzymes involved in DNA replication, RNA transcription, recombination, and purine assembly. Specific functions of enzymes that fell within the secondary processes category were secondary metabolite production (e.g., nonribosomal peptide synthases, polyketide synthetases, and RTX toxins) (Marahiel et al. 1997; Hur et al. 2012; Hamdache et al. 2013), virulence (e.g., Rhs family proteins and adhesins), extracellular sensing (e.g., histidine kinases), and helicases. Large, extracellular, alpha-helical proteins, which includes proteins such as horizontally acquired alpha-2-macroglobulins that are typically found in pathogenic or saprophytically colonizing species and likely assist in colonization (Budd et al. 2004), comprised, along with uncharacterized membrane proteins, the majority of large bacteroidial proteins. Additionally, Rhs family proteins were among the most abundant large proteins missing in OIEs of either phylum. These poorly characterized proteins have been implicated in inflammasome activation in *Pseudomonas* (Kung et al. 2012) and involved in signaling peptide transport across the inner membrane (Hill et al. 1994). Conserved hypothetical proteins of unknown function were also abundant among the largest nonOIE proteins and absent in OIE proteomes. The absence of large proteins in OIE proteomes can be explained by the loss of lifestyle-specific proteins, present as individual loci or as genomic islands (i.e., Frank et al. 2002), due to relaxed selection for these functions under the constant, intracellular host conditions. Alternatively, there might have been strong selection for the loss of these large accessory proteins early in genome reduction (Lee and Marx 2012), although the cost of having these large proteins for OIEs is not clear. Obviously, deletion of long genes nonessential for intracellular habitation and mutualism with the host will contribute to rapid genome reduction more so than loss of shorter genes, but it is not clear, nor is it within the scope of this study to examine, why genes encoding enzymes involved in core cellular processes were generally shorter than those of unknown function or involved in secondary cellular processes.

### Conclusion

Protein lengths do not scale with genome reduction as expected, but genetic drift and relaxed selection explain the observed length variations in OIE orthologs. As additional genomes for obligate insect endosymbionts from other bacterial phyla become available, expansion of this study will be possible to ascertain the robustness of our results across diverse bacterial lineages. This work sets the stage for further characterization of the impact of the indels observed in OIE orthologs on their functions. In light of observed increased constitutive GroEL expression in *Buchnera* (Fares et al. 2004), indels in endosymbiont orthologs may have led to decreased protein stability that GroEL overexpression may compensate for. Alternatively, indels in endosymbiont orthologs may impact their substrate-binding capacities, leading to promiscuous binding and possible additional functional capabilities (i.e., Kelkar and Ochman 2013), representing adaptations of endosymbionts to different host lifestyles and/or their massive gene losses, but additional experimental analyses are required. Finally, genome-wide relaxed selection for genes in OIE genomes, which has lead to an observed increased ortholog length variation, also contributes to rapid accumulation of inactivating mutations in loci tangential to the intracellular lifestyle. These mutations eventually lead to loss of these genes, potentially with little consequence to the mutualism, and contribute to overall genome reduction.

## Supplementary Material

Supplementary materials S1–S10 are available at *Genome Biology and Evolution* online (http://www.gbe.oxfordjournals.org/).

Supplementary Data
