# Supplementary material for: Obligate Insect Endosymbionts Exhibit Increased Ortholog Length Variation and Loss of Large Accessory Proteins Concurrent with Genome Shrinkage
Source: Genome Biol Evol. 2014 Mar 26;6(4):763–75. doi: 10.1093/gbe/evu055 (PMC4007534; doi:10.1093/gbe/evu055)
Supplement: Supplementary Data [file supp_6_4_763__index.html]

Obligate insect endosymbionts exhibit increased ortholog length variation and loss of large accessory proteins concurrent with genome shrinkage. — Obligate Insect Endosymbionts Exhibit Increased Ortholog Length Variation and Loss of Large Accessory Proteins Concurrent with Genome Shrinkage — Supplementary Data 

# Obligate Insect Endosymbionts Exhibit Increased Ortholog Length Variation and Loss of Large Accessory Proteins Concurrent with Genome Shrinkage

## Supplementary Data

files

**Files in this Data Supplement:**

- Supplementary Data - pdf file
- Supplementary Data - pdf file
- Supplementary Data - pdf file
- Supplementary Data - pdf file
- Supplementary Data - pdf file
- Supplementary Data - pdf file
- Supplementary Data - xlsx file
- Supplementary Data - xlsx file
- Supplementary Data - docx file
- Supplementary Data - docx file
- Supplementary Data - xlsx file
- Supplementary Data - xlsx file
