# Supplementary figures and images for: Obligate Insect Endosymbionts Exhibit Increased Ortholog Length Variation and Loss of Large Accessory Proteins Concurrent with Genome Shrinkage
Source: Genome Biol Evol. 2014 Mar 26;6(4):763–75. doi: 10.1093/gbe/evu055 (PMC4007534; doi:10.1093/gbe/evu055)

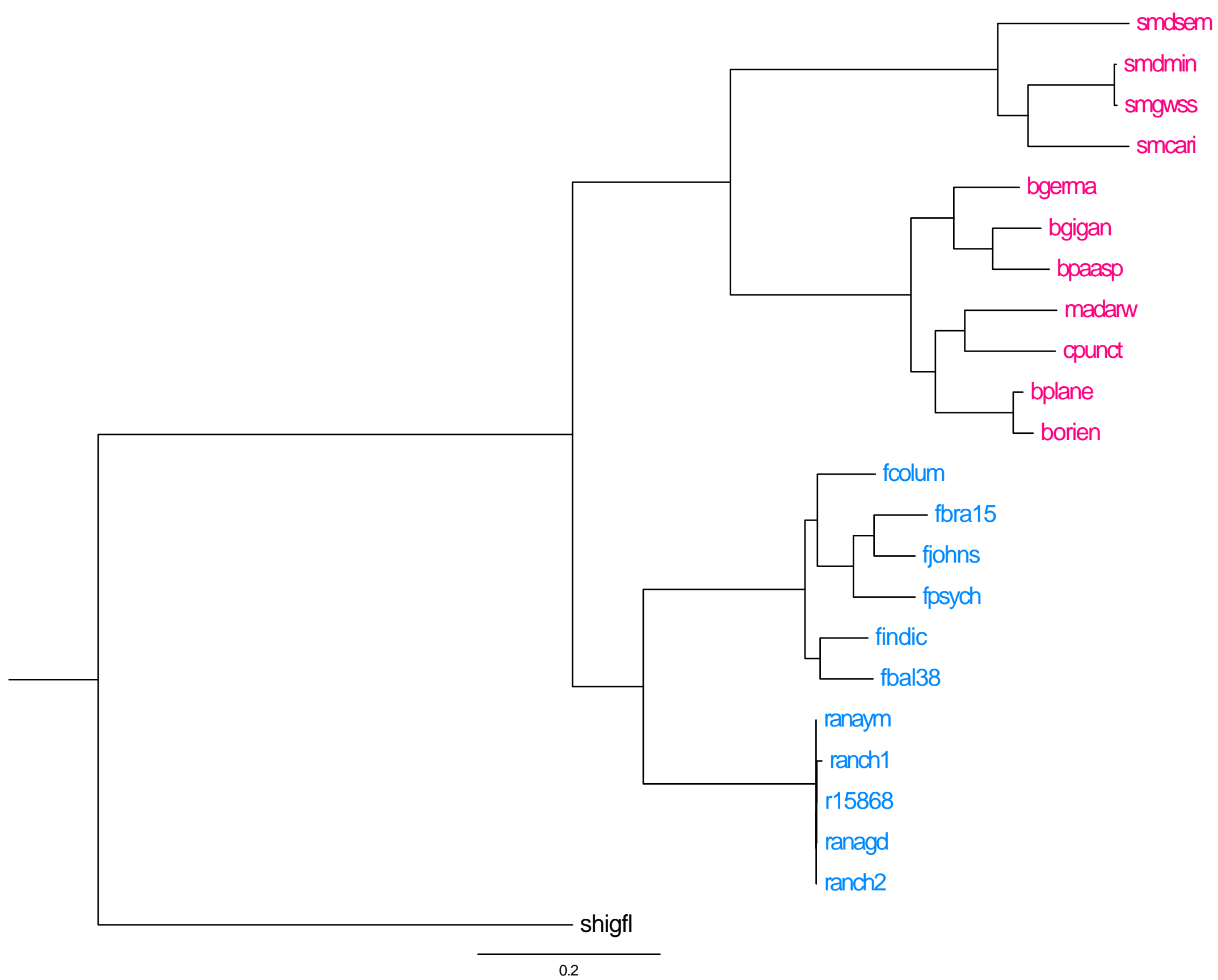

Supplement: Supplementary Data [file supp_evu055_Supplementary_Materials_S2a.pdf]

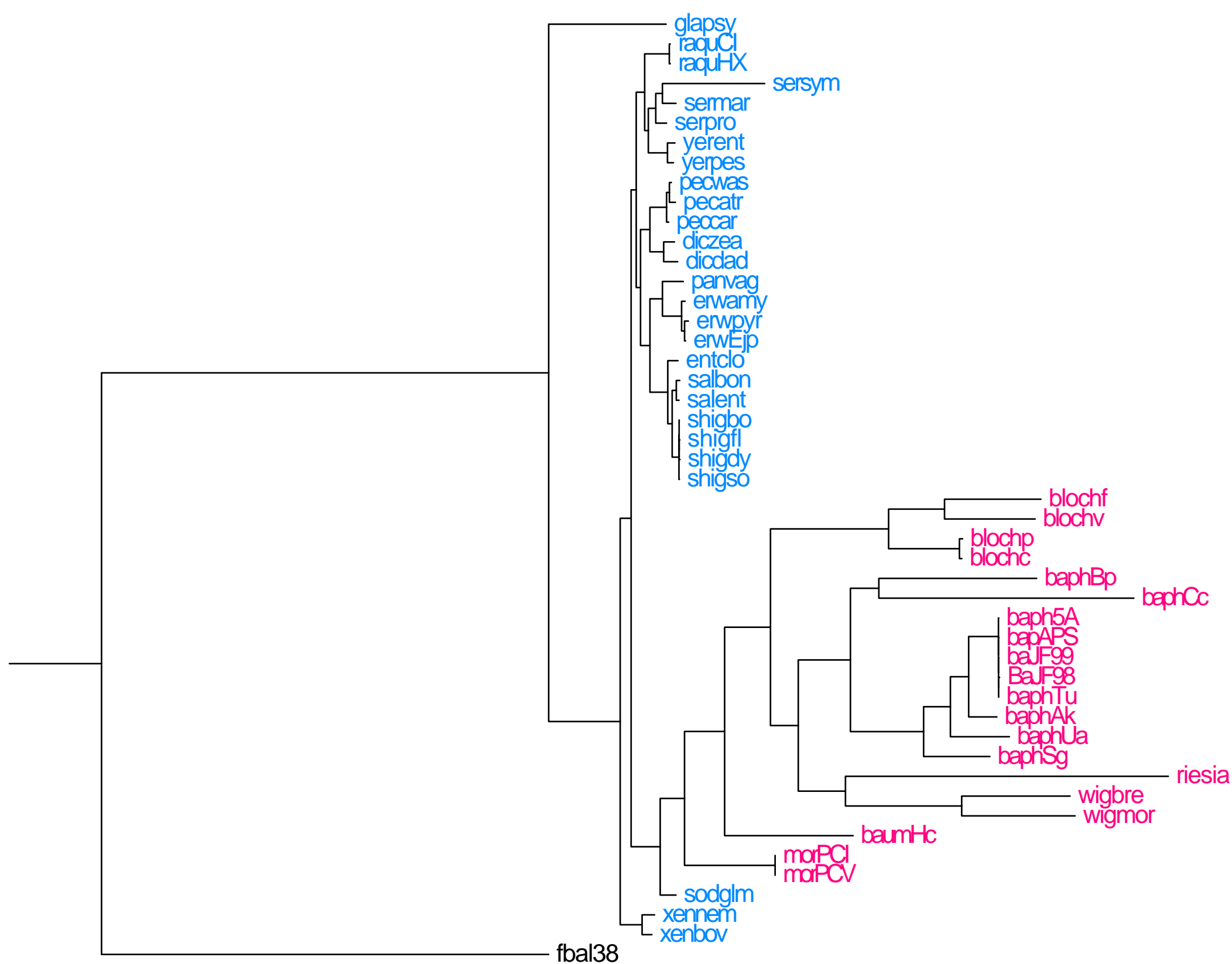

0.3

Supplement: Supplementary Data [file supp_evu055_Supplementary_Materials_S2b.pdf]

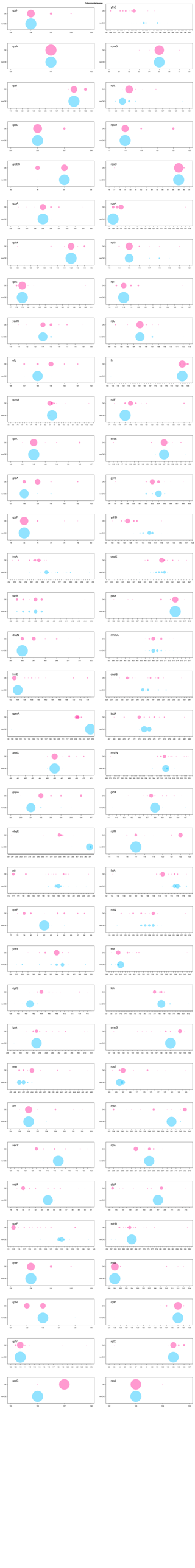

Supplement: Supplementary Data [file supp_evu055_Supplementary_Materials_S3a.pdf]

# Flavobacteriaceae

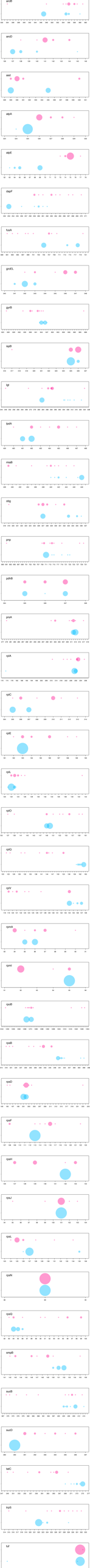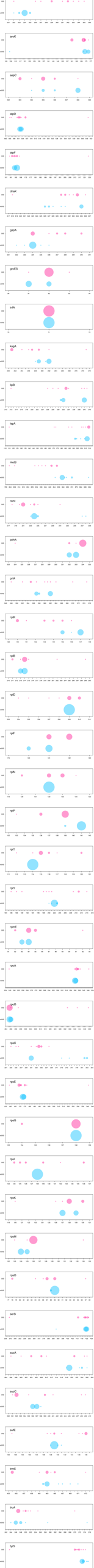

Supplement: Supplementary Data [file supp_evu055_Supplementary_Materials_S3b.pdf]

### *Enterobacteriaceae*

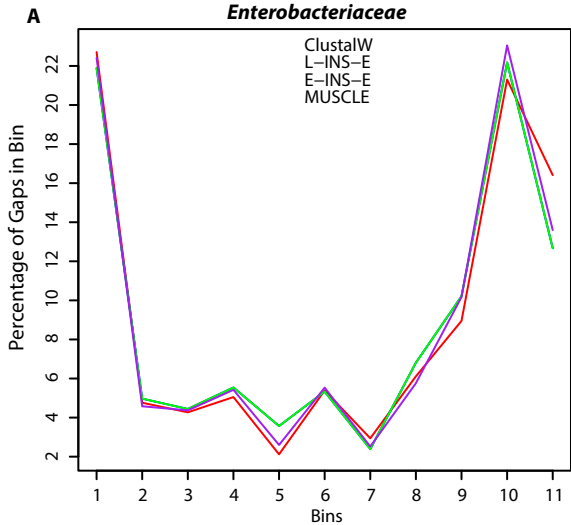

### *Flavobacteriaceae*

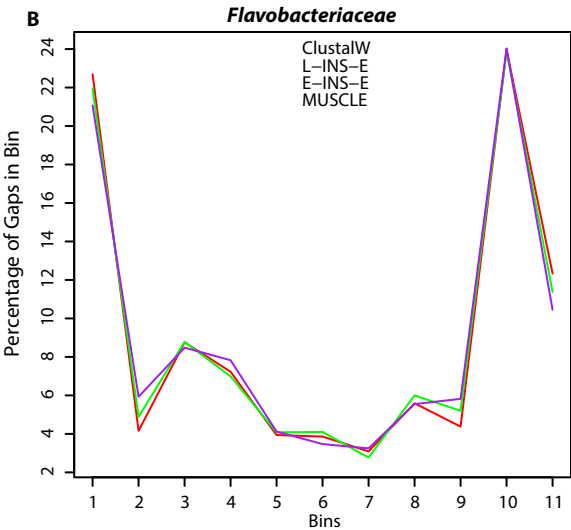

Supplement: Supplementary Data [file supp_evu055_Supplementary_Materials_S9.pdf]
