# Supplementary material for: Obligate Insect Endosymbionts Exhibit Increased Ortholog Length Variation and Loss of Large Accessory Proteins Concurrent with Genome Shrinkage
Source: Genome Biol Evol. 2014 Mar 26;6(4):763–75. doi: 10.1093/gbe/evu055 (PMC4007534; doi:10.1093/gbe/evu055)
Supplement: Supplementary Data [file supp_evu055_Supplementary_Materials_S8.pdf]

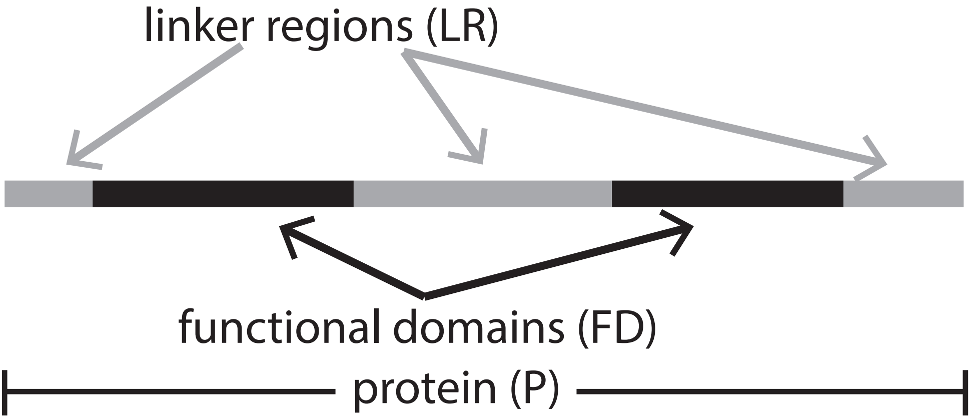

λ = # amino acids

$$\Sigma FD_{\lambda} = FD1_{\lambda} + FD2_{\lambda}$$

$$\Sigma LR_{\lambda} = P_{\lambda} - \Sigma FD_{\lambda}$$
