# Supplementary material for: Obligate Insect Endosymbionts Exhibit Increased Ortholog Length Variation and Loss of Large Accessory Proteins Concurrent with Genome Shrinkage
Source: Genome Biol Evol. 2014 Mar 26;6(4):763–75. doi: 10.1093/gbe/evu055 (PMC4007534; doi:10.1093/gbe/evu055)
Supplement: Supplementary Data [file supp_evu055_Supplementary_Materials_S4.docx]

**Supplementary Materials S4:** Average standard deviations for all orthologous protein groups in each family and ANOVAs comparing standard deviations across orthologous protein groups.

|  | **Flavobacteriaceae** | **Enterobacteriaceae** |
| --- | --- | --- |
| **Proteins** | 6.22 | 3.50 |
| **Domains** | 4.78 | 2.34 |
| **Linkers** | 5.95 | 3.42 |
| **ANOVA P-value** | 0.226 | 0.134 |
