# Supplementary material for: Obligate Insect Endosymbionts Exhibit Increased Ortholog Length Variation and Loss of Large Accessory Proteins Concurrent with Genome Shrinkage
Source: Genome Biol Evol. 2014 Mar 26;6(4):763–75. doi: 10.1093/gbe/evu055 (PMC4007534; doi:10.1093/gbe/evu055)
Supplement: Supplementary Data [file supp_evu055_Supplementary_Materials_S5.docx]

**Supplementary Materials S5:** Standard deviations (“StDev”) of lengths of proteins shared between Flavobacteriaceae and Enterobacteriaceae.

|  | **Enterobacteriaceae** | | **Flavobacteriaceae** | |
| --- | --- | --- | --- | --- |
|  | *OIE* | *nonOIE* | *OIE* | *nonOIE* |
| **Average StDev** | 2.83 | 0.77 | 5.25 | 1.49 |
| **T-Test p-value** | 4.38E-06** | | 0.0009** | |
